# Large area kidney imaging for pre-transplant evaluation using real-time robotic optical coherence tomography

**DOI:** 10.1038/s44172-024-00264-7

**Published:** 2024-09-02

**Authors:** Xihan Ma, Mousa Moradi, Xiaoyu Ma, Qinggong Tang, Moshe Levi, Yu Chen, Haichong K. Zhang

**Affiliations:** 1 Department of Robotics Engineering, Worcester Polytechnic Institute, Worcester, MA USA; 2 Department of Biomedical Engineering, University of Massachusetts, Amherst, MA USA; 3 The Stephenson School of Biomedical Engineering, University of Oklahoma, Norman, OK USA; 4 Department of Biochemistry and Molecular & Cellular Biology, Georgetown University, Washington, DC USA; 5 College of Photonic and Electronic Engineering, Fujian Normal University, Fuzhou, Fujian PR China; 6 Department of Biomedical Engineering, Worcester Polytechnic Institute, Worcester, MA USA

**Keywords:** Biomedical engineering, Optical imaging

## Abstract

Optical coherence tomography (OCT) can be used to image microstructures of human kidneys. However, current OCT probes exhibit inadequate field-of-view, leading to potentially biased kidney assessment. Here we present a robotic OCT system where the probe is integrated to a robot manipulator, enabling wider area (covers an area of 106.39 mm by 37.70 mm) spatially-resolved imaging. Our system comprehensively scans the kidney surface at the optimal altitude with preoperative path planning and OCT image-based feedback control scheme. It further parameterizes and visualizes microstructures of large area. We verified the system positioning accuracy on a phantom as 0.0762 ± 0.0727 mm and showed the clinical feasibility by scanning ex vivo kidneys. The parameterization reveals vasculatures beneath the kidney surface. Quantification on the proximal convoluted tubule of a human kidney yields clinical-relevant information. The system promises to assess kidney viability for transplantation after collecting a vast amount of whole-organ parameterization and patient outcomes data.

## Abstract (web summary)

Xihan Ma and colleagues expand the field-of-view for optical coherence tomography using a robotic manipulator to control the probe. They achieve high position precision in ex vivo demonstration.

## Introduction

The global shortage of suitable organs has led to a backlog of patients with end-stage renal diseases (ESRD) who are awaiting kidney transplantation^1^. This situation is largely due to the lack of a reliable means to assess the viability of available kidneys for transplant^2^, which results in a high discard rate of donor organs^3^. Quantitative evaluation of deceased donor kidneys is commonly performed using the Kidney Donor Profile Index (KDPI)^2^, derived from the donor’s medical history, and the pathological scoring^4–6^ from pre-transplant donor biopsy (PTDB). Nonetheless, the correspondence between KDPI and the success of the transplant has not yet been definitively established^7–12^. On the other hand, PTDB is an invasive procedure with restricted sampling volume that can introduce bias to the organ scoring^13–15^. A recent comparative study on four commonly used biopsy scoring systems shows none of them is strongly correlated with post-transplant graft survival or early graft function^16^. Therefore, there is a strong need for alternative pre-transplant kidney evaluation methods to be developed for predicting future graft function that could help minimize organ discards on deceased-donor kidneys.

Optical coherence tomography (OCT) is a well-established imaging modality capable of providing high-resolution, in situ, and real-time 2D cross-sectional imaging (B-scans) of biological samples^17^. Recent studies have demonstrated the ability of OCT to provide non-invasive histopathological information about the kidney^18–21^. Moreover, pre-transplant OCT imaging of kidneys has been shown to enable the prediction of post-transplant renal function^22^. Such prediction can be made by evaluating the morphology of the proximal convoluted tubule (PCT) lumen structures^22,23^. Hence, incorporating OCT-based scoring as an adjunct evaluation step to the already widely adopted KDPI and PTDB methods could enhance the reliability of the organ quality evaluation outcome.

Unlike PTDB, OCT imaging is non-invasive and contactless, thus it is feasible to scan over the entire kidney surface and create a spatially-resolved score map in order to minimize the risk of getting a biased OCT-based organ quality evaluation. However, this is challenging because most of the reported OCT systems in literature only provide sub-centimeter level narrow lateral field-of-view (FOV) while maintaining a high lateral resolution (e.g., around tens of microns)^24–26^, largely due to mechanical constraints posted on the OCT beam sweep angle^27^. Unfortunately, such a limited FOV is inadequate to capture a regular sized human kidney (~120 mm by ~50 mm^28^). While the emerging miniaturized hand-held OCT systems^29,30^ allow scanning wider area by positioning the OCT probe at multiple places, the functionality to precisely track the probe in free space is absent, making it difficult to spatially correspond B-scans with the sample. It is, therefore, necessary to investigate new OCT imaging systems capable of wider area imaging and accurate probe localization.

Various attempts have been made on developing novel OCT instrumentation in the pursuit of enlarging OCT imaging FOV and enabling spatially resolved OCT images. These instruments differ from each other in terms of formfactors, which can be categorized into (i) custom-built OCT system with improved light source sweeping mechanism, (ii) OCT system where the probe is attached with spatial tracking device for freehand scanning, and (iii) OCT system where the probe is robotically actuated for constrained and tracked scanning. An example of formfactor (i) was developed by Song et al. where akinetic swept source and wide-angle camera lens were employed in the self-built tabletop OCT system to expand the FOV of conventional OCT^31^. The system achieved ultra-fast (a few seconds) large FOV (200 by 200 mm) imaging, yet resulting in relatively lower resolution. However, pre-transplant kidney assessment is a resolution-sensitive task. Moreover, their system still requires manual focusing depending on the size of the sample to be imaged, which might become cumbersome during the clinical procedure. Therefore, the customized system is not best suited for kidney imaging. For (ii), Qin et al. presented a hand-held OCT probe^32^ which offers unconstrained FOV and the ability to image samples in situ. The probe is tracked in six degree-of-freedom (DoF) using visual-odometry (VO) techniques^33^ through an RGB-depth camera mounted on the probe which perceives the probe’s surrounding environment. Yet, the VO algorithm’s localization accuracy depends heavily on the detection of trackable environmental features, hence, is not robust across different in-door conditions. To address the drawbacks of formfactor (ii), robotic OCT (R-OCT) systems (formfactor (iii)) providing large FOV with accurate scanning motion and consistent localization accuracy have been investigated extensively in recent years. Göb et al. integrated an OCT system with a Cartesian robot platform for large area vascular contrast skin imaging^34^. Nonetheless, the developed translational stage has restricted range of motion and probe manipulability. Draelos et al. introduced R-OCT system^35,36^ using a six DoF robotic arm for OCT probe positioning. The system can follow human head motion in real-time and track the human eye for autonomous ophthalmology OCT exam. Huang et al. presented an R-OCT^37^ that scans large tissue and generates wide FOV 3D OCT volume via stitching spatially connected small FOV volumes which were acquired by a 3D OCT probe at multiple predefined locations. Their follow-up work by Li et al. focused on the automated scan path planning using a structure light camera^38^. The aforementioned volume stitching principle was then applied in other R-OCT systems by He et al. for in vivo whole mouse brain OCT angiography^39^ and Göb et al. for large area skin imaging^40^.

The above previous works have showed possibilities to extend the OCT imaging FOV and the usability of large area OCT scans in various biomedical applications. Among these formfactors, we believe the R-OCT solution possesses unique advantage over the others when it comes to biological tissue imaging as it provides sufficient flexibility to sample placement while being able to produce stable, repeatable scanning motion.

However, the volume stitching strategy adopted in most previous R-OCT systems has a practical challenge in clinical kidney imaging. Due to the tradeoff between the OCT’s lateral resolution and confocal parameter (depth of field)^26^, when the sample surface is highly uneven (such as in the case of a human kidney), even a small FOV volume is likely to contain suboptimal-quality B-scans where the sample is beyond the depth of field. More importantly, an OCT system with extended FOV that can perform high resolution imaging of a regular human kidney (~100 mm × 50 mm), with a specific aim to visualize renal microstructures has not been reported in the literature.

Herein, we introduce a different solution to the volume stitching based R-OCT systems where 2D OCT images are continuously acquired along the scanning trajectory. This solution was first presented in 2022^41^ to address large area OCT imaging for transplant kidney evaluation and was later adopted and further verified by Li et al. to enable large area scan of various biological samples^38^. However, in addition to the 2D image acquisition, we further leveraged the established real-time communication between the OCT system and the robot controller to continuously acquire 2D OCT images and dynamically keeps the scanning sample within the depth of field by optimizing the OCT probe’s position (x, y, z) per B-scan. This approach provides advantages over the previous works as it guarantees consistent quality of the B-scans, allowing the system to handle organs with highly steep surface. Furthermore, our system offers flexibility in the scanning time since the rate of the robot motion is largely adjustable. Such flexibility allows for quick coverage of the tissue, hence is clinically valuable. The system’s imaging accuracy and the ability to scan the whole kidney are validated on customized phantom and kidney samples ex vivo. Finally, we demonstrate fully automated large area kidney parameter mappings showing clinically relevant kidney tissue heterogeneity, which has not been shown (or has to be performed manually^37^) in the previous research.

## Results

### R-OCT for large area imaging

**Fig. 1:**
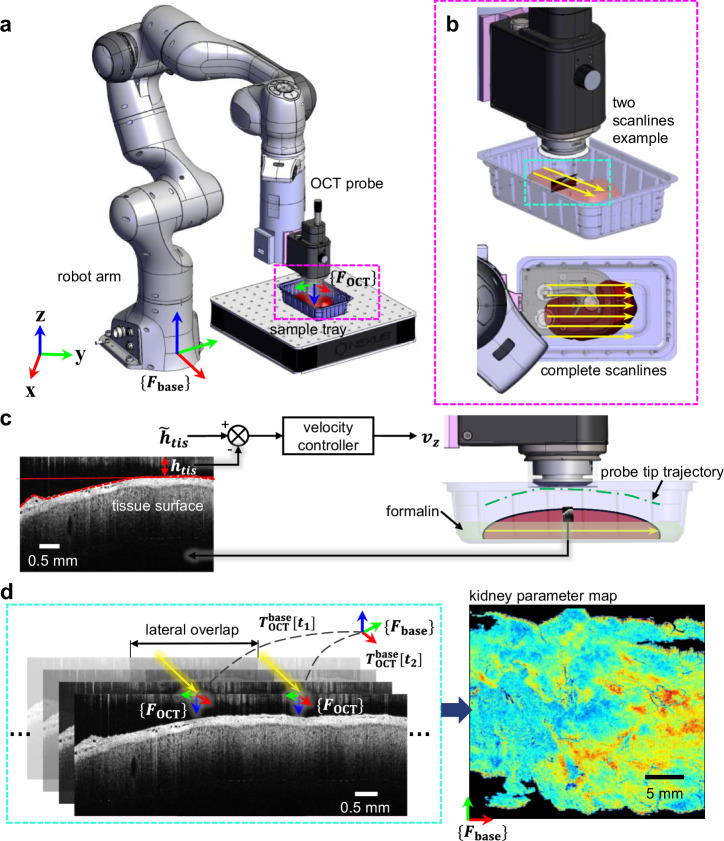
The Robotic Optical Coherence Tomography (R-OCT) system for large area imaging. **a** R-OCT system layout and coordinate system definition. $\{{F}_{{base}}\}$ is the robot arm’s base coordinate frame; $\{{F}_{{OCT}}\}$ is the Optical Coherence Tomography (OCT) probe coordinate frame, originated at the top center of the OCT B-scan. **b** Zoomed-in views of the sample placement region. A kidney sample to be imaged lies inside the sample tray. Yellow arrows represent scanlines. An example B-scan is rendered along the scanlines (top). **c** OCT probe adjustment scheme using real-time B-scans as control feedback. **d** post-processing steps after the large area scan completion. ${T}_{{OCT}}^{{base}}$ is the homogeneous coordinate transformation from $\{{F}_{{base}}\}$ to $\{{F}_{{OCT}}\}$. ${T}_{{OCT}}^{{base}}[{t}_{1}]$ is the transformation recorded from the robot pose measurement at timestamp ${t}_{1}$; ${T}_{{OCT}}^{{base}}[{t}_{2}]$ is the transformation recorded at ${t}_{2}$ along an adjacent scanline. The OCT B-scans are first spatially localized (left), then parameterized and reprojected for large-area 2D visualization (right).

Our R-OCT system^41^ consists of a seven DoF robotic arm (Panda, Franka Emika, Germany) and a customized end-effector which houses a compact OCT probe (OCTG13 Telesto, Thorlabs, USA). The spectral-domain OCT system has a center wavelength of 1325 nm with an incident power of 2.5 mW. The probe has a 36 mm focal length (LSM03, Thorlabs Inc., USA) objective, offering 13 μm lateral and 5.5 μm axial resolution in air. The system was configured to provide a lateral resolution of 2.73 μm per pixel and axial resolution of 2.68 μm per pixel, respectively. Two workstations, communicating through wireless network, are used for robot motion control and OCT data acquisition respectively. Adopting multiple workstations alleviates the computation burden posed on each end, hence minimizing the chance of receiving unexpected system delay during imaging. Further, this design choice allows the large system to be decoupled for better extendibility (e.g., different imaging modalities^42,43^ may be involved for future needs). During imaging, the robot performs multiple straight-line motion with respect to the robot base frame, $\{{F}_{{base}}\}$ (referred to as scanlines) to cover the entire sample (see Fig. 1b). The scanlines, generated prior to the imaging, are defined by a few manually entered parameters including a starting point based on sample placement, the length of each scanline, the lateral distance to be covered by the scanlines and the amount of offset between consecutive scanlines such that the OCT images are overlapped laterally (see more details “Methods”). While traveling through a scanline, OCT images are streamed and recorded at 20 frames-per-second (fps) with an image size of 1800 pixels (lateral) by 700 pixels (axial). Based on the depth of automatically detected tissue surface in the B-scans, the altitude of the probe is adjusted in real-time to compensate for the uneven surface so that the image quality is consistent. After the scan, all collected OCT images and their corresponding probe poses are archived. To enable intuitive interpretation of the large area imaging outcomes, a series of spatially-resolved parameterization methods are developed to extract and display anatomical information from the spatially localized OCT images. These visualization methods include: (i) depth-encoded mapping (DEPM) for capturing the surface curvature of the sample; (ii) axial-attenuation mapping (ATCM) for revealing microstructures beneath the tissue surface; and (iii) PCT lumen diameter mapping (DIAM) for extracting and quantifying specific clinically relevant information. The implementation details on the generation of these mappings will be discussed in the “Methods”. As a brief technical overview, DEPM is obtained by projecting the z-axis coordinate of kidney surface to the x-y plane of the robot base frame; ATCM is obtained in the same projection plane, but by projecting the rate of light attenuation, estimated using a single scattering model; DIAM, on the other hand, projects the binary mask of PCT lumen detected in OCT B-scans by leveraging a deep learning based segmentation network. In the following results, we mainly used DEPM to validate our R-OCT system’s probe localization accuracy. The ATCM and DIAM are used to generate large area parameterizations revealing the anatomical structure under the tissue surface for potential pathological analysis. Note that the above visual representations are all in 2D. This is mainly for the consideration of (i) computational efficiency as a 3D rendering of the kidney’s microstructures in a large area could result in inefficient use of memory and graphics resources, and (ii) the user intuitiveness as the organ visualized in 2D can be understood by the user at one glance without the need for adjusting the viewing angle. However, generating and rendering the 3D OCT volume of a local area where dense anatomy can be visualized without costing too much computation resource is possible. Readers can refer to Supplementary Fig. S1 for representative volume renders.

### Imaging 3D printed phantom

**Fig. 2:**
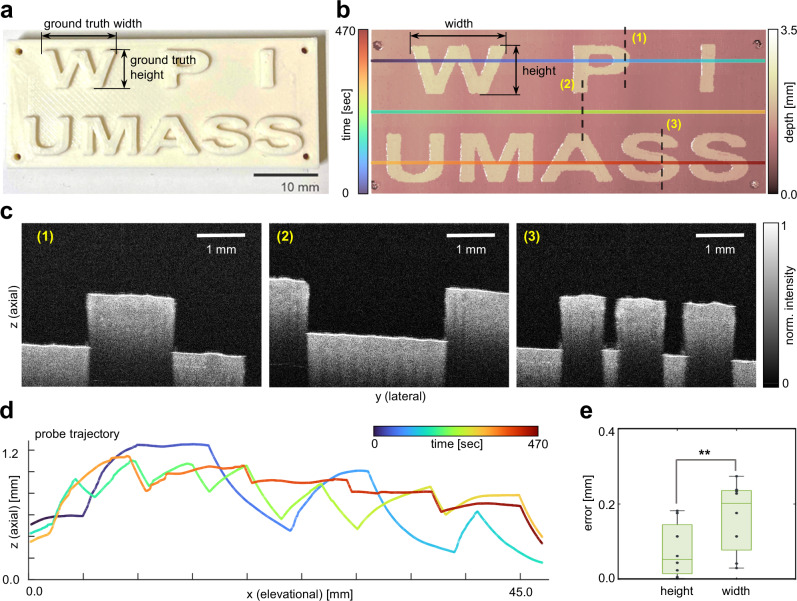
Imaging 3D printed phantom. **a** Picture of the 3D printed phantom. The phantom has eight letter-shape extrusions (W, P, I, U, M, A, S, S). **b** reconstructed depth-encoded mapping (DEPM) of the 3D printed phantom overlaid by the (Optical Coherence Tomography) OCT probe scan trajectory. **c** Representative OCT B-scans from imaging the phantom. The location of these images relative to the sample can be found in **b**. **d** The OCT probe scan trajectory viewed from the axial-elevational plane. **e** Box plot of the errors in restoring the height and width of the letters in DEPM. The central mark in each plot indicates the median. The top and bottom edges of the boxes represent the 25^th^ and 75^th^ percentile, respectively. The top and bottom bars indicate the maximum and minimum error values. N=8 in each category. P=0.021 (student’s t-test)

The R-OCT system’s probe localization accuracy plays a vital role in enabling large area spatially-resolved OCT scan. To verify the localization accuracy, a specifically designed phantom, fabricated through 3D printing (48 mm by 20 mm) was imaged by the R-OCT system. As shown in Fig. 2a, the phantom has eight extrusions in the form of English letters. Three scanlines were pre-programmed to achieve complete coverage of the phantom (1.5 mm lateral overlap between consecutive scanlines). During the imaging process, the robot traveled continuously at a speed of 0.35 mm s^−1^ along each scanline, resulting in a total imaging time of 470 s (including the time to store the OCT images). The distance between the OCT probe and the phantom surface was automatically regulated by controlling the probe’s vertical position to ensure optimal image quality (see “Methods”). The effectiveness of the automatic probe adjustment can be observed through the consistent phantom surface position in the B-scans and the probe trajectory displayed in the sideview (see Fig. 2c, d, respectively). It is worth noting that when imagining the 3D printed phantom, the letter extrusions appear suddenly in the OCT image, posing extra challenge on the responsiveness of the automatic probe adjustment. Therefore, the probe adjustment tends to yield better performance when imaging biological sample of regular surface (e.g., kidney sample). Individual B-scans were spatially aligned using the probe localization information for large-area visualization. We used DEPM of the detected phantom surface to visualize the texture on the phantom (see “Methods”). The phantom DEPM, which depicts a reconstruction of the letters, is presented in Fig. 2b. The pixel resolution of the DEPM is 2.73 μm per pixel in lateral and 21.0 μm per pix in elevational direction. It can be shown that the R-OCT system successfully covers the entire footprint of the phantom, capturing all eight letter extrusions, which demonstrates its capability for large-area imaging. Such a wide scan area is unattainable with existing tabletop OCT imagers. Furthermore, minimum distortions were found in the shape of the letters, indicating a high degree of probe positioning accuracy. The probe localization accuracy was quantified by comparing the width and height of the letters measured from the phantom DEPM against the same measurements performed directly on the phantom with a caliper. The average errors in letter reconstruction were found to be 0.0762 ± 0.0727 mm in height and 0.1670 ± 0.0941 mm in width (see Fig. 2e). While the overall accuracy is acceptable, a student’s t-test concluded that the error in the height of the reconstructed letters is significantly smaller than the error in the width of the letters (P = 0.021). This means that the DEPM has higher precision in the lateral direction than in the elevational direction (~0.1 mm difference). Further analysis on the heterogeneity of the probe tracking accuracy in different directions will be added in the “Discussion” section.

### Imaging ex vivo porcine kidney sample

**Fig. 3:**
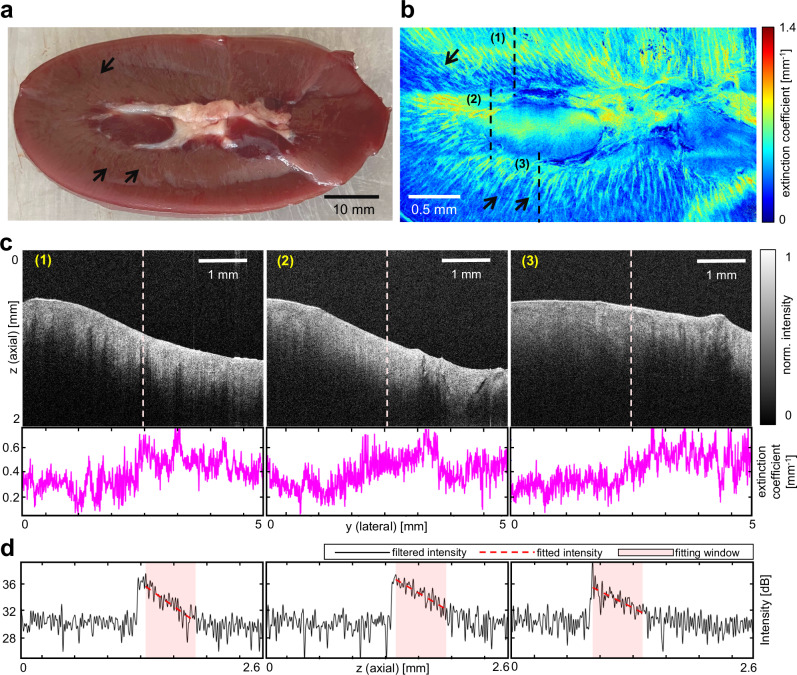
Imaging ex-vivo porcine kidney. **a** Picture of the porcine kidney sample. **b** Axial-attenuation mapping (ATCM) reconstruction of the kidney sample. **c** Representative optical coherence tomography (OCT) B-scans from imaging the kidney and the per-column (A-scan) extinction coefficient values for each image. The location of these images relative to the sample can be found in **b**. **d** center column A-scan of the images in **c** (marked by white dotted lines).

To illustrate the R-OCT system’s capability of visualizing kidney microstructures, an ex vivo study was conducted using a porcine kidney sample with a dimension of 70.8 mm by 50.1 mm as the imaging target. To enhance the observation of well-known renal anatomy, including the renal cortex and medulla, the kidney sample was cut open with a scalpel (see Fig. 3a). The imaging procedure followed a similar protocol in the previous phantom experiment, covering an area of 44 mm by 21 mm. At the visualization step, instead of solely extracting the sample surface, spatially aligned B-scans were further parameterized using the extinction coefficient, calculated per vertical line (A-scans) in the B-scans. The utilization of extinction coefficient has been demonstrated to effectively differentiate tissue by their optical attenuation properties^44–46^, thereby facilitating the visualization of the kidney microstructures. Figure 3c showcases several representative B-scans along with the corresponding extracted extinction coefficients. The ATCM parameterization technique compresses 2D B-scans into 1D vectors, enabling large-area 2D ATCM visualization (see “Methods” section). Figure 3b shows the porcine kidney ATCM with pixel resolution of 2.73 μm per pixel in lateral and 29.7 μm per pixel in elevational direction. The ATCM reveals the presence of radially distributed straight lines, particularly in the periphery of the ATCM (indicated by the arrows). Similar landmarks can be seen in the photograph of the sample in Fig. 3a. These landmarks can be corresponded to characteristic anatomical features of renal medulla^47^, as the renal medulla consists of a series of renal pyramids that exhibits striations due to the presence of straight tubular structures and blood vessels. This finding proves that the ATCM is able to show kidney morphological features under the surface.

### Imaging ex vivo human kidney sample

**Fig. 4:**
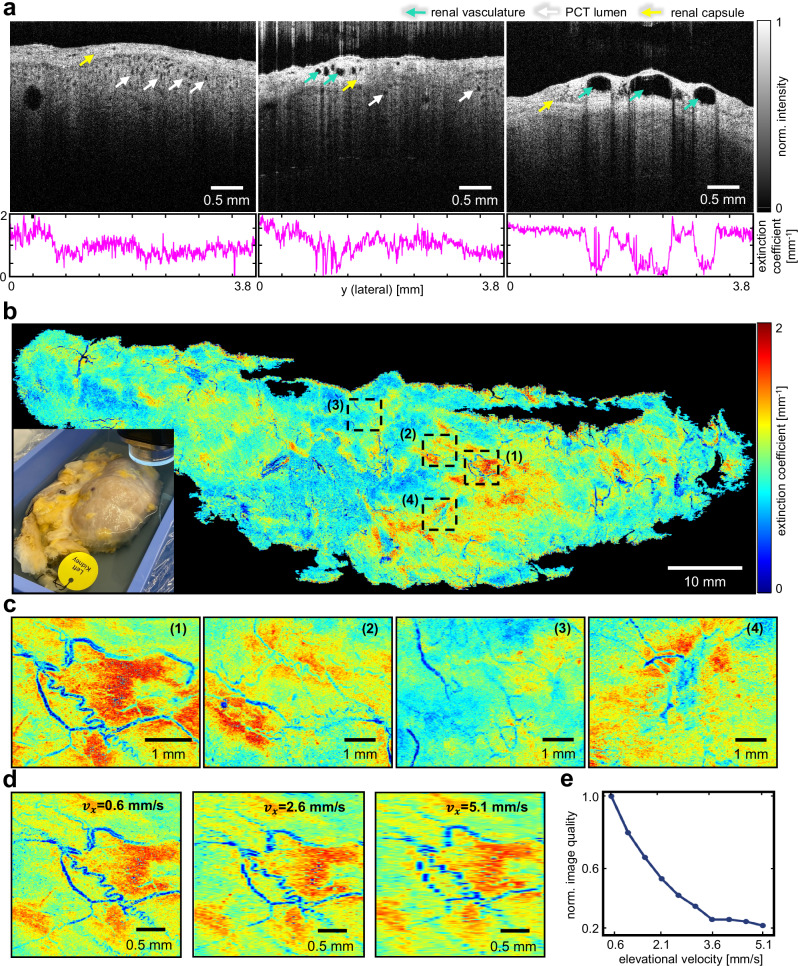
Imaging ex-vivo human kidney. **a** Representative B-scans acquired from the human kidney sample, along with the per-A-scan extinction coefficient values for each image. **b** The whole kidney axial-attenuation mapping (ATCM) and a picture of the kidney sample (bottom left). **c** Four regions of interest (ROI) zoomed in from **c** (ROI 1-4). **d** ROI 1 downsampled in elevation direction at different intervals to simulate altered robot scan velocities. **e** Simulated ATCM reconstruction quality change caused by altering the robot scan speed to N=10 different values.

In kidney transplantation, the donor kidney needs to be well-protected and examined in situ. Therefore, unlike the open-cut porcine kidney experiment, the imaging of the human kidney was restricted to the organ’s surface without damaging the tissue. To validate the R-OCT system under a realistic situation, an intact ex vivo human kidney with dimensions of 106.39 mm by 37.70 mm was imaged by our system. The kidney underwent vascular perfusion to keep the key anatomies from collapse before being preserved in formalin (see “Methods”). During imaging, only the surface of the kidney was exposed to air while the bottom portion remained submerged in formalin (see bottom left in Fig. 4b). Ten scanlines were employed to ensure comprehensive coverage of the kidney (3 mm lateral overlap between consecutive scanlines). The Supplementary video demonstrates the complete scanning of the human kidney.

Figure 4a illustrates three representative B-scans along with their corresponding extinction coefficient vectors, revealing the presence of multiple identifiable anatomical landmarks, including the renal capsule, the capsular perforating radiate arteries^47^, and the PCT lumen. The perforating radiate arteries are discernible circular structures exhibiting low extinction coefficient values. Overall, the scan covered an area of 123.61 mm by 53.41 mm, sufficiently covering the footprint of the kidney sample. The whole kidney ATCM (2.73 μm per pixel lateral resolution and 29.7 μm per pixel elevational resolution, extra areas were cropped), which depicts vessel-shaped microstructures at multiple places on the kidney surface was generated in Fig. 4b. Four regions of interest (ROI) within the whole kidney ATCM were magnified to enable detailed visualization of the fine vasculatures (Fig. 4c). These vasculatures are identified as the perforating arteries located within the renal capsule due to their diameter of around 0.15 mm.

Note that the data to produce the ATCM in Fig. 4b was collected with the robot traveling at 0.6 mm s^−1^ in the elevational direction, resulting in a total imaging time of 2700 s (including data storage time). In clinical practice, however, the time spent on OCT data collection is expected to be minimized to reduce the exposure of the sample in air. To investigate the effect of faster robot scanning speed on the ATCM, an additional study was conducted where the ATCM was downsampled at different intervals to simulate different robot scanning velocities. The intervals were computed given a robot velocity range from 0.6 mm s^−1^ to 5.1 mm s^−1^ with an increment of 0.5 mm s^−1^, while maintaining a fixed B-scan acquisition rate (20 fps). The maximum speed of 5.1 mm s^−1^ was selected to accelerate the scan process by 8.5 times in theory, compressing entire procedure to 317 seconds, i.e., 5 min. In practice, this time is expected to be further shortened as the data storage will also be accelerated (scanning at 0.6 mm s^−1^ results in 40,920 B-scans in total. This number is expected to be reduced to 4,814 when scanning at 5.1 mm s^−1^). Presumably, the ATCM would suffer from distortions when the robot traveled at higher speeds. The distortions can be quantified by the reduction in the image quality of ATCM, which is computed using the structure similarity metric^48^ between the downsampled ATCM and the original ATCM (normalized). The degradation in the image quality as the robot travels faster can be observed in Fig. 4d. It can be summarized from Fig. 4e. that the degradation exhibited an exponential trend as the robot’s speed increased, reaching a plateau around 0.25 which corresponds to a speed of 3.6 mm s^−1^.

**Fig. 5:**
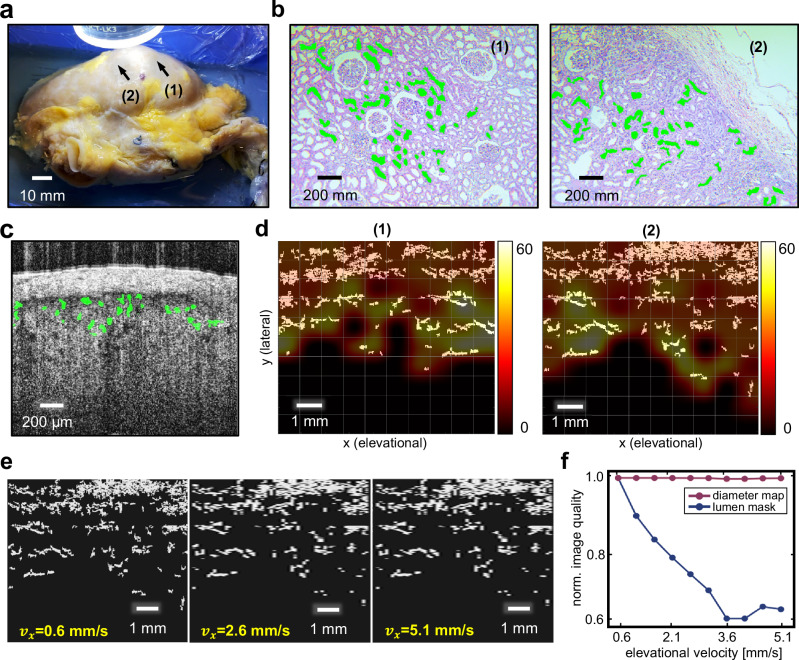
Large area parameterization of the ex vivo human kidney. **a** Picture of the human kidney sample. Two representative region of interests (ROIs), labeled as (1) and (2) were used to generate proximal convoluted tubule lumen diameter mapping (DIAM) (each ROI is ~ 8 mm by 8 mm). **b** Post-scan histopathology of the two ROIs. Green masks are the manually labeled tubule structures identified in the microscopic picture. **c** Representative proximal convoluted tubule (PCT) lumen segmentation in a B-scan acquired from the human kidney sample. The green masks the detected PCT. **d** Segmented PCT lumen in binary mask, viewed from the top, overlaid by the DIAM for ROI (1) and (2). **e** PCT lumen mask in ROI (2) downsampled in elevation direction at different intervals to simulate the altered robot scan velocities. **f** Simulated lumen mask and DIAM quality change caused by altering the robot scan speed to N=10 different values.

Though the whole kidney ATCM can effectively show renal vasculatures within the renal capsule, its sensitivity to PCT lumens with diameters less than ~30 μm is limited. However, our previous work has indicated a high correlation between the morphology of the PCT lumens and the occurrence of the delayed graft function (DGF)^22^. As DGF is a critical factor affecting the kidney transplant outcomes, it is necessary to detect the PCT lumens explicitly in B-scans and generate comprehensive parameter maps for lumen diameter to provide direct quantitative organ assessment. For this purpose, we employed a deep learning based semantic segmentation model^49^ for automatic PCT lumen detection per B-scan. Figure 5c illustrates a representative B-scan overlaid with binary lumen segmentation mask. By spatially aligning these segmentation masks, a large area 3D volume containing segmented PCT lumen can be constructed (white clusters in Fig. 5d). The DIAM was then generated subsequently (see “Methods”). The colormap overlay in Fig. 5d represents the DIAM for two illustrative ROIs whose locations on the kidney sample were labeled in Fig. 5a. The average lumen diameter for the ROIs were measured to be 21.28 ± 19.75 μm for ROI (1) and 23.29 ± 17.88 μm for ROI (2) from the DIAM. The relatively larger standard deviation in the OCT measurements may be stemmed from errors in the automatic lumen segmentation across a large number of frames (300 slices per ROI). To further validate the diameter values, we extracted tissue from these two ROIs by slicing from roughly the same plane for OCT imaging to perform post-scan histopathological analysis. The microscopic pictures for the tissue samples were shown in Fig. 5b (one picture per ROI). The tubule structures were manually labeled with green mask whose average diameters were measured to be 22.38 ± 4.56 μm for ROI (1) and 20.67 ± 4.87 μm for ROI (2). The average diameter values show a close match across the two modalities.

To examine the change in the mask volume and DIAM, the same robot velocity sweep experiment was conducted. Figure 5e highlights the change of the top-viewed mask volume as the scan speed increased. Similarly, the distortion in these maps was evaluated using the image quality measure. As shown in Fig. 5f, the mask volume deteriorated rapidly at a higher scan speed. However, little change was captured in the DIAM, showing the level of robustness of these two visualization methods when the elevational imaging resolution changes.

## Discussion

In summary, we have developed an R-OCT system that performs large sample imaging for pretransplant kidney quality assessment. We showcased the system’s ability to achieve large FOV imaging (theoretically covering a half-circular region of radius 855 mm) which is nonviable with existing OCT imaging techniques. The reconstruction of the phantom texture using DEPM showcased the system’s sub-millimeter level probe localization accuracy, as well as the probe altitude adjustment ability to deal with the steepness of the sample surface. The whole kidney comprehensive scan shows the potential to apply the R-OCT system in a real application scenario. Furthermore, the large area parameter map offers intuitive visualization on the anatomies under the kidney surface. The porcine kidney ATCM reveals microstructures that can be correlated with the sample photography. The DIAM for the human kidney shows lumen diameter values similar to the numbers reported in the literature^22^. The mean diameter of the lumen structure measured under OCT shows a close match with those measured under microscopic pictures for both ROI (1) and ROI (2). These results demonstrated the feasibility of using the presented large area parameter maps to extract clinically relevant information.

Compared to handheld OCT probes, our R-OCT system shows distinct superiorities in terms of providing stable scan motion and precise probe localization. Unlike most previous R-OCT systems presented in the literature that stitches small FOV OCT volumes to achieve wider area scan, our system features synchronized acquisition of OCT B-scans and their corresponding OCT probe poses. This feature enables us to optimize the probe altitude (see “Methods”) based on per-sectional imaging feedback in real-time. Therefore, our system can better adapt to tissues with high surface variability such as a human kidney (the altitude difference in our human kidney sample reached 23.6 mm, way beyond typical OCT penetration depth of ~2 mm). In case of imaging renal margin with steep surface, we can increase the overlapped area between consecutive scanlines to make sure that the tissue remains in the OCT field of depth. In addition, it eliminates the need for prior information about the sample geometry in order to determine a substantial number of volume acquisition points (our system only requires specifying two points in elevational and lateral directions as the start and the end to generate a scanline). Nor does the system need to orthogonally reorient the probe towards the tissue according to an initial rough volume acuquisition^37^, As a result, the overall imaging workflow becomes more efficient and less sensitive to inaccurately defined trajectory. Lastly, while we did not encounter any unexpected robot motion due to noisy OCT B-scans, it might be worth exploring advanced and robust tissue identification techniques^38^ to add extra reliability to the system.

However, one drawback associated with the 2D image-based acquisition is that spatial alignment of the OCT images may suffer from lower accuracy in the elevational direction than in the lateral direction, which can be seen from the letter reconstruction result in the phantom imaging experiment. This discrepancy arises because elevational reconstruction accuracy relies solely on the recorded OCT probe pose measured by robot joint motor encoders. In contrast, the reconstruction in the lateral direction depends less on the probe pose measurements but more on the lateral FOV of the B-scans, which inherently provides higher accuracy. To mitigate this issue, scanning the sample bi-directionally (i.e., generating scanlines in orthogonal directions) could be employed, albeit at the cost of prolonging the imaging procedure.

Nevertheless, an unneglectable advantage of our system is the flexibility to adjust the elevational imaging resolution along the scanline direction by altering the robot scan speed. Such flexibility can be utilized by the clinicians to perform quick scans on the organ to receive a general impression on the organ healthiness. Although fewer images can be acquired at a higher scan speed, leading to less elevational pixel resolution and potentially distorted anatomies (see Figs. 4c and 5e), the DIAM is shown to be minimally affected by the increased scan speed (Fig. 5f). Therefore, they are ideal visualization tools that can be leveraged to provide an overall assessment of the organ. Next, several smaller regions showing suspicious lesion or clinically interested landmarks can be further inspected with dense imaging at a lower scan speed. Such workflow has been implemented in other imaging tasks such as breast ultrasound examination^50^.

For future directions, the software pipeline for OCT data acquisition and OCT-to-robot communication will be further optimized to reach faster B-scan sampling rate, such that the imaging time can be shortened, and higher elevational pixel resolution can be achieved. An automatic sample recognition scheme using depth camera^37,38^ will be incorporated to eliminate the need for manual parameter tuning when generating the scan trajectory. In addition, we will work on establishing an imaging protocol to integrate the current system into the clinical workflow for pretransplant kidney monitoring by maximally utilizing the scan time flexibility of the presented R-OCT system. The protocol is expected to detail the optimal robot scanning speed, overlapped area among scanlines, the sterilization workflow, etc. to make the overall imaging time and outcome meet the clinical expectations. Owing to the limited number of accessible kidney samples, only one human kidney was scanned. However, being able to differentiate diverging quality kidneys with large area OCT based scoring is important to confirm the clinical significance of the parameter maps (ATCM and DIAM). To this end, an increased number of samples involving both high- and low-quality kidneys will be imaged by our R-OCT and compared against histopathological ground truth.

Although the R-OCT system is mainly demonstrated for kidney imaging, we envision such a platform is generalizable to other anatomies such as skin^40^ and brain^51^, as well as other applications such as cancer imaging (ATCM has been used in differentiating tumor from normal tissues^52^). Alternative tissue analysis can be conducted using, for instance, deep-learning based reasoning for tumor diagnosis procedure^53^. It is also worth noting that the robotic arm features superior dexterity and accuracy in positioning the OCT probe compared to hand-held^32^ and translational stage solutions^34^. In the presented experiments, we mainly employed pure translational scan motion to ensure consistent optical attenuation across the tissue for ATCM generation. Nevertheless, the capability of probe adjustment based on OCT image feedback makes it possible to also rotate the probe orthogonally with respect to the sample surface in real-time to prevent defocusing. Such dexterity suggests our platform can be potentially used intraoperatively for surgical navigation^54,55^ by providing real-time, multi-location tissue tomography. However, it is important to note that the presented study involves no tissue motion as the in vitro kidney sample was placed in a static container. Additional implementation on tissue motion detection and compensation is necessary to enable in vivo applications using R-OCT.

## Methods

### OCT and robotic system integration

The Spectral Domain OCT (SD-OCT) system used in this paper (1300 nm central wavelength) has been previously used for similar kidney monitoring applications^22^. The robotic arm was designed for applications involving human interaction. Its seven DoF allows more generalized use case of the platform (see “Discussion”). Two desktop workstations were used for robot motion control (referred to as PC1) and OCT image acquisition (referred to as PC2). Real-time communication between PC1 and PC2 was facilitated by the Robot Operating System (ROS), (Stanford Artificial Intelligence Laboratory et al. (2018). *Robotic Operating System*. Retrieved from https://www.ros.org), allowing the utilization of OCT images as feedback for the adjustment of probe altitude. The communications delay between the two workstations is roughly measured to be 6 ms. The rigid body transformation ${T}_{{{{\rm{OCT}}}}}^{{{{\rm{base}}}}}\in {SE}(3)$, which represents the transformation from the robot base frame $\{{F}_{{{{\rm{base}}}}}\}$ to the OCT probe frame $\{{F}_{{{{\rm{OCT}}}}}\}$, consists of two separate transformations, ${T}_{{{{\rm{flange}}}}}^{{{{\rm{base}}}}}$ and ${T}_{{{{\rm{OCT}}}}}^{{{{\rm{flange}}}}}$. ${T}_{{{{\rm{flange}}}}}^{{{{\rm{base}}}}}$ is the transformation from $\{{F}_{{{{\rm{base}}}}}\}$ to the last link of the arm which is obtained from the real-time robot pose measurement. ${T}_{{{{\rm{OCT}}}}}^{{{{\rm{flange}}}}}$ is the transformation from the last link to $\{{F}_{{{{\rm{OCT}}}}}\}$, calibrated based on the CAD model of the customized end-effector attachment. The robot was controlled via sending Cartesian velocity commands of $\{{F}_{{{{\rm{OCT}}}}}\}$ relative to $\{{F}_{{{{\rm{base}}}}}\}$ at a rate of 1000 fps using the Franka Control Interface (FCI) programmed in C + + language. The OCT images were streamed to PC2 using the Spectral Radar’s (Thorlabs, USA) MATLAB (MATLAB, MathWorks, USA) SDK, where temporal filtering was applied to reduce the OCT noise to establish robust image feedback control. The acquired images were stored for later post-processing, i.e., generating the parameter maps.

### R-OCT large area scan procedure

With the sample positioned at a designated region within the sample tray, the robot initiates its operation from a predefined home configuration. To achieve full-coverage of the sample, a set of pre-coded scanlines is utilized. These scanlines are distributed along the y-axis of $\{{F}_{{{{\rm{base}}}}}\}$ and points to the x-axis direction; each scanline begins at the same starting point in the x-axis and z-axis directions of $\{{F}_{{{{\rm{base}}}}}\}$ with a consistent length. Formally, they can be characterized by a set of parameters: $\{{x}_{{{{\rm{st}}}}},{y}_{{{{\rm{st}}}}},{z}_{{{{\rm{st}}}}},L,W,{W}_{{{{\rm{ol}}}}}\}$, where $\{{x}_{{{{\rm{st}}}}},{y}_{{{{\rm{st}}}}},{z}_{{{{\rm{st}}}}}\}\in {{\mathbb{R}}}^{3\times 1}$ represents the starting coordinate under $\{{F}_{{{{\rm{base}}}}}\}$ for the first scanline; $L{\mathbb{\in }}{\mathbb{R}}$ denotes the length for all scanlines (i.e., the distance to be covered in the x-axis of $\{{F}_{{{{\rm{base}}}}}\}$); $W{\mathbb{\in }}{\mathbb{R}}$ is the distance to be covered in the y-axis direction; ${W}_{{{{\rm{ol}}}}}{\mathbb{\in }}{\mathbb{R}}$ is the desired overlap between consecutive B-scans from adjacent scanlines. Among these parameters, *L* and *W* are obtained by measuring the size of the sample; ${W}_{{{{\rm{ol}}}}}$ is empirically tuned; $\{{x}_{{{{\rm{st}}}}},{y}_{{{{\rm{st}}}}},{z}_{{{{\rm{st}}}}}\}$ is determined by manually dragging the OCT probe to a desired location with the robot set to the teaching mode. Note that the teaching process only needs to be done once as the sample will always be placed at the designated region. Different sample sizes can be accommodated by manually adding relative offsets to the starting coordinate. The total number of scanlines, denoted as $n$, can be calculated as follows:

1 $$n=\left\lceil \frac{W}{{W}_{{{{\rm{OCT}}}}}-{W}_{{{{\rm{ol}}}}}},\right\rceil s.t.\, {W}_{{{{\rm{OCT}}}}}\, > \,2{W}_{{{{\rm{ol}}}}}\, > \,0$$

where ${W}_{{{{\rm{OCT}}}}}{\mathbb{\in }}{\mathbb{R}}$ is the lateral FOV of the OCT probe. The process of traversing through the $i$-th ($i\in [1,n]$) scanline comprises three steps.

Step1. At initial time ${t}_{0}$, the robot first positions the probe at an entry pose, ${T}_{{{{\rm{OCT}}}}}^{{{{\rm{base}}}}}[{t}_{0}]$, which expanded to:

2 $${T}_{{{{\rm{OCT}}}}}^{{{{\rm{base}}}}}\left[{t}_{0}\right]=\left[\begin{array}{cccc}1 & 0 & 0 & {x}_{{{{\rm{st}}}}}\\ 0 & -1 & 0 & {y}_{{{{\rm{st}}}}}+\left(i-1\right)\left({W}_{{{{\rm{OCT}}}}}-{W}_{{{{\rm{ol}}}}}\right)\\ 0 & 0 & -1 & {z}_{{{{\rm{st}}}}}\\ 0 & 0 & 0 & 1\end{array}\right]$$

At ${z}_{{{{\rm{st}}}}}$, the probe is sufficiently distant from the sample in the vertical direction, such that the sample does not appear in the OCT image.

**Step 2**. Without altering the orientation of the probe with respect to $\{{F}_{{{{\rm{base}}}}}\}$, a landing motion is executed, moving the probe vertically towards the sample at a constant velocity along the z-axis of $\{{F}_{{{{\rm{base}}}}}\}$. When the OCT probe approaches the sample closely enough, the surface of the sample can be detected in the OCT image with straightforward intensity thresholding technic incorporating an upper and lower bound to reduce the chance of recognizing surface reflection as the true tissue surface. The position of the sample inside the image is quantified by the normalized surface depth (NSD), *μ*, defined as:

3 $${{\mu }}=1-\frac{{h}_{{{{\rm{tis}}}}}}{{H}_{{{{\rm{OCT}}}}}},s.t.\,{H}_{{{{\rm{OCT}}}}} \, > \, {h}_{{{{\rm{tis}}}}}\, > \, 0$$

where ${{{{\rm{H}}}}}_{{{{\rm{OCT}}}}}$ is the axial FOV of the OCT image; ${{{{\rm{h}}}}}_{{{{\rm{tis}}}}}$ corresponds to the depth of the highest point on the detected tissue surface in the B-scan (depicted in Fig. 1c). The sample surface should be positioned reasonably close to the probe to maintain focus yet allowing some distance to avoid the tissue from exceeding the OCT axial FOV or attaching the probe tip. To this end, a threshold NSD (*μ* = 0.75) is empirically determined as the termination condition for the landing motion, i.e., when *μ* ≥ 0.75.

Step 3. The robot moves the probe at a constant speed, ${v}_{{{{\rm{x}}}}}$, along the scanline (in x-axis), capturing B-scans in real-time while recording the probe pose at every time stamp. Throughout the imaging process, the depth of the sample surface in the B-scans (i.e., *μ*) is regulated via adjusting the probe altitude to ensure consistent image quality. Details regarding the surface depth regulation, referred to as NSD regulation, will be discussed in the next subsection. The robot halts once the probe has traveled a distance of $L$, indicating the completion of one scanline. The probe is then repositioned at the starting point of the next scanline and the same process will be repeated until the last scanline is completed.

### Online probe altitude adjustment

As mentioned above, the NSD regulation is employed using a proportional feedback controller that outputs linear velocity along the z-axis of $\{{F}_{{{{\rm{base}}}}}\}$ based on the current OCT image. At timestamp $t$, the probe velocity along the z-axis of $\{{F}_{{{{\rm{base}}}}}\}$, ${v}_{{{{\rm{z}}}}}[t]$, is given by:

4 $${v}_{{{{\rm{z}}}}}\left[t\right]={w}_{{{{\rm{s}}}}}{K}_{{{{\rm{p}}}}}\left(\widetilde{{{\mu }}}-{{\mu }}\right)+\left(1-{w}_{{{{\rm{s}}}}}\right){v}_{{{{\rm{z}}}}}[t-1]$$

where ${w}_{{{{\rm{s}}}}}\in [{{\mathrm{0,1}}}]$ is the low-pass filter weight to avoid velocity jittering, such that the robot motion is less vulnerable to incorrect and inconsistent tissue surface detection; ${K}_{{{{\rm{p}}}}}$ is the velocity gain; $\widetilde{{\mu }}$ is the desired NSD (empirically set to 0.75); *μ* is the NSD computed from the current image; With the velocity controller, the tissue will be maintained at a fixed depth, which can be verified by the example B-scans shown in Figs. 2c, 3c and 4a. In addition to the velocity regulation, the absolute position of the probe altitude is also constrained within a certain range (the range was determined prior to the scan based on the sample positioning) so that the probe is unlikely to collide into the tissue or other obstacles.

### Large area 2D parameter map generation

Here we explain the process to generate post-scan large area parameterized visualization, including DEPM, ATCM and DIAM using the spatially tracked OCT images. For each scanline, the process can be divided into two steps, namely, step 1, which entails generating a large area volume using localized OCT images; and step 2, which involves parameterizing 2D slices of the volume and reprojecting the parameterized volume for 2D visualization.

Step 1 further comprises two sub-steps: (i) all OCT images are transformed into a common static coordinate frame ($\{{F}_{{{{\rm{base}}}}}\}$), and (ii) the voxelization of the images. The pose of the $i$-th OCT image relative to the robot base, collected at timestamp $t$, is denoted as ${T}_{{{{\rm{OCT}}}}}^{{{{\rm{base}}}}}[{t}_{i}]$. For each pixel in the image, its coordinate ${{{{\bf{p}}}}}^{{{{\rm{OCT}}}}}\in {{\mathbb{R}}}^{3}$ under $\{{F}_{{{{\rm{OCT}}}}}\}$ can be transformed to $\{{F}_{{{{\rm{base}}}}}\}$ through the equation below (coordinates are augmented for homogeneous matrix multiplication):

5 $${{{{\bf{p}}}}}^{{{{\rm{base}}}}}={T}_{{{{\rm{OCT}}}}}^{{{{\rm{base}}}}}[{t}_{i}]\left({{{{\bf{p}}}}}^{{{{\rm{OCT}}}}}\cdot {{{{\boldsymbol{\alpha }}}}}_{{{{\rm{res}}}}}\right)$$

where ${{{{\bf{p}}}}}^{{{{\rm{base}}}}}$ represents the coordinate under $\{{F}_{{{{\rm{base}}}}}\}$; ${{{{\boldsymbol{\alpha }}}}}_{{{{\rm{res}}}}}\in {{\mathbb{R}}}^{3}$ is the pixel resolution (pixel per mm). Applying this operation to all the images yields the spatially aligned OCT images. Second, these images are voxelized by discretizing the pixel coordinates under $\{{F}_{{{{\rm{base}}}}}\}$, resulting in a large area 3D tissue volume, noted as ${{{{\mathcal{V}}}}}_{{{{\rm{tis}}}}}$.

Step 2 involves varying parameterization techniques depending on the specific visualization purposes. For DEPM generation, the depth of the sample surface in each slice of ${{{{\mathcal{V}}}}}_{{{{\rm{tis}}}}}$ is extracted using pixel intensity thresholding and projected from the top view (x-y plane of $\{{F}_{{{{\rm{base}}}}}\}$).

In the case of ATCM, each A-scan in every slice of ${{{{\mathcal{V}}}}}_{{{{\rm{tis}}}}}$ is parameterized using the extinction coefficient (${\mu }_{{{{\rm{t}}}}}$). The value of ${\mu }_{{{{\rm{t}}}}}$ is derived by fitting the single scattering model^44^ to the A-scan within a given depth window. The single scattering model is written as:

6 $$I\left(z\right)\propto \exp (-2{\mu }_{{{{\rm{t}}}}}\cdot z)$$

where $I\left(z\right)$ is the pixel intensity at the imaging depth $z$ in the depth window. The depth window is limited to close to the sample surface (~ 1 mm) because most meaningful anatomical features only appear near-surface. Example intensity fitting results can be found in Fig. 3d. Lastly, the ATCM can be formed by spatially aligning ${\mu }_{{{{\rm{t}}}}}$.

The DIAM generation requires first segmenting the PCT lumens from ${{{{\mathcal{V}}}}}_{{{{\rm{tis}}}}}$ to construct a binary mask volume. To accomplish this, we first perform contrast enhancement^56^ on each slice of ${{{{\mathcal{V}}}}}_{{{{\rm{tis}}}}}$. The preprocessed slices are fed into a Residual-Attention-UNET model previously developed^49^ to obtain segmentation masks for PCT lumen. This model, pretrained on 14,403 OCT images, achieved an accuracy of 0.82 in terms of Dice index and 0.85 in terms of Intersection over Union (IoU) metric on its original testing dataset. In this work, however, we did not perform a separate segmentation accuracy evaluation as the process involves vast amount of expert-annotated images and benchmarking the lumen segmentation accuracy is not the main purpose of our paper. Instead, the PCT lumen diameters are calculated and compared with the histopathology ground truth which also proves the correctness of the segmentation. The resulting mask volume then undergoes a series of slice-by-slice morphological reconstructions (i.e., area closing followed by area opening operation) to ensure the shape consistency of the lumen segmentation mask. The output binary volume, ${{{{\mathcal{V}}}}}_{{{{\rm{msk}}}}}$, is viewed from the top perspective. Next, we calculate the lumen diameter for each slice of ${{{{\mathcal{V}}}}}_{{{{\rm{msk}}}}}$ using the centerline algorithm^32^. The top-view projection of ${{{{\mathcal{V}}}}}_{{{{\rm{msk}}}}}$ is then downsampled into $k$-by-$k$ grids. Within each grid, the diameter of the segmented lumens that fall inside are averaged. Finally, the $k$-by-$k$ grid map is upsampled to its original size via bi-cubic interpolation. In our case, $k$ is empirically assigned to be 10.

After obtaining the parameter map from each scanline, the parameter maps are stitched together using the recorded probe poses. Parameter maps from adjacent scanlines are averaged in the overlapped area to form a large area parameter map with smooth transition between scanlines.

### Kidney sample preparation and post-scan histopathology

The fresh porcine kidney sample was obtained from a local slaughterhouse. The experiment-used human kidney sample was obtained from a deceased donor who did not meet the transplant standard. It was preserved by hypothermic machine perfusion (HMP) for keeping the kidney sample’s physiological status and then fixed by 10% formalin within 2 days after removing from the donors. This study was approved by the University of Oklahoma and the University of Oklahoma Health Sciences Center Institutional Review Board (IRB) (Study number: IRB #12462).

The post-scan histopathology was performed at the University of Massachusetts Chan Medical School, Worcester, Massachusetts, USA. The sliced kidney samples were processed with H&E staining and imaged with a pre-calibrated microscope with 10× magnification.

### Statistics and reproducibility

The 3D printed phantom was scanned two times by the R-OCT system using identical imaging parameters. No major differences were observed in the outcome across different scans. The ex-vivo porcine kidney and the ex-vivo human kidney were scanned only one time to minimize the sample exposure to air. Prolonged exposure may lead to deteriorated sample surface. Therefore, no reproducibility statement can be concluded through this study.

### Reporting summary

Further information on research design is available in the Nature Portfolio Reporting Summary linked to this article.

## Supplementary information

Supplementary material

Description of Additional Supplementary Files

Supplementary Video 1

Reporting summary

## Data Availability

The data that support the findings of this study are available from the corresponding author upon reasonable request.

## Code availability

The software to generate large area kidney tissue parameter maps is developed using MATLAB, tested under MATLAB 2022b. The deep learning based PCT lumen segmentation is developed using Python 3.8. All scripts are available from the corresponding author upon reasonable request.
