# Supplementary material for: Large area kidney imaging for pre-transplant evaluation using real-time robotic optical coherence tomography
Source: Commun Eng. 2024 Sep 2;3:122. doi: 10.1038/s44172-024-00264-7 (PMC11368928; doi:10.1038/s44172-024-00264-7)
Supplement: Supplementary file 1 — Supplementary material [file 44172_2024_264_MOESM1_ESM.pdf]

Supplementary Information for

Local Area Volume Rendering of Ex-vivo Human Kidney Sample

This file includes:

Supplementary Figure 1

Supplementary Notes on Volume Rendering Results

Other Supplementary Material for this manuscript includes the following:

Supplementary video

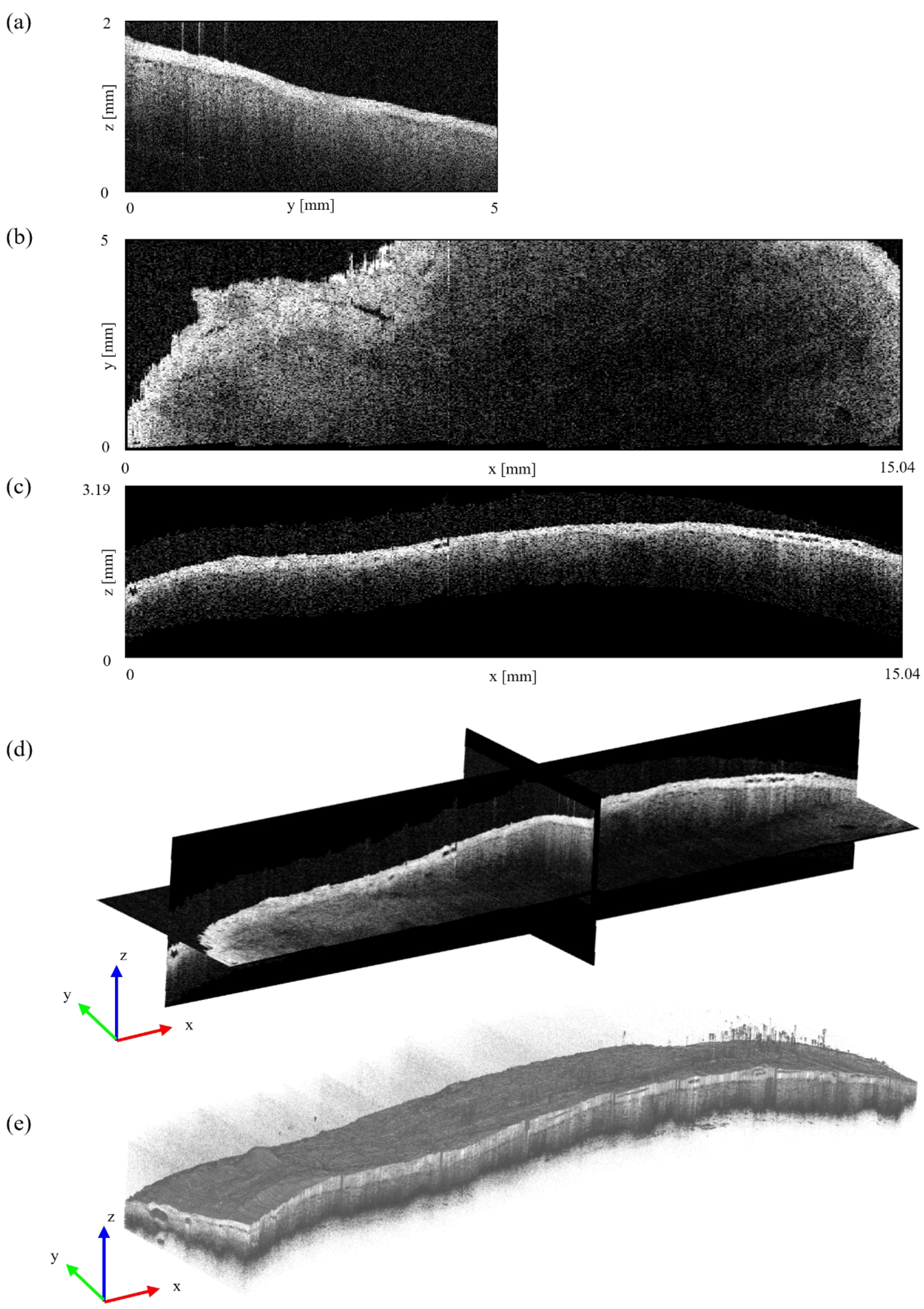

Supplementary Figure 1. Local 3D kidney volume visualization. (a) axial-lateral slice plane. The red bounding box highlights the anatomy (PCT lumens) captured; (b) lateral-elevational slice plane; (c) axial-elevational slice plane; (d) spatially aligned slicing planes. (e) 3D rendering of the local volume.

Supplementary Notes

- **Supplementary Note 1**

While the main context of the manuscript only showed 2D large area parameterizations of the kidney (i.e., DEPM, ATCM and DIAM), it is also possible to reconstruct 3D volume since the OCT B-scans are spatially tracked. Here we show the 3D volume reconstruction and rendering results of a local area on the ex-vivo human kidney (same sample used in Fig. 4-5 in the manuscript) to confirm that the OCT B-scans are spatially tracked by the robotic arm with good accuracy. The selected local area is of size 5.00 mm x 3.19 mm x 15.04 mm (lateral x axial x elevational). The reason for choosing a local area for 3D visualization is because the altitude variation of the kidney surface is small enough to present dense, high-resolution, and memory-efficient volume rendering. It is noteworthy that the 15.04 mm elevational field of view has significantly surpassed what typical desktop OCT system can offer.

We spatially aligned the OCT B-scans according to the time stamped OCT probe poses to generate the local area volume. **Supplementary Figure 1** shows the slice plane views and the 3D rendering of the local volume. The slice planes and the volume are shown with respect to the robot base frame (see the manuscript for details).

It can be observed that many kidney microstructures, including the proximal convoluted tubule (PCT) lumens are captured by the local volume. However, it is difficult for the radiologists to quickly identify the anatomy of interest at the first glance of the kidney volume. Therefore, in the manuscript, we choose to use different visualization methods rather than the 3D volume rendering for the display of clinically relevant information.
