# Supplementary material for: Large area kidney imaging for pre-transplant evaluation using real-time robotic optical coherence tomography
Source: Commun Eng. 2024 Sep 2;3:122. doi: 10.1038/s44172-024-00264-7 (PMC11368928; doi:10.1038/s44172-024-00264-7)
Supplement: Supplementary file 2 — Description of Additional Supplementary Files [file 44172_2024_264_MOESM2_ESM.pdf]

Description of Additional Supplementary Files

File name: Supplementary Video 1

Description: The supplementary video contains the recording of the proposed R-OCT system scanning an ex-vivo human kidney (left). Real-time OCT images are streamed and recorded along with the OCT probe poses (top right). A volume rendering of a local area of the kidney sample reveals fine vessel structures, showing accurate OCT probe localization (bottom right).
